# Self-Reported Patient Compliance With Physician Advised Lifestyle Behavior Changes Among Adults With Musculoskeletal Conditions

**DOI:** 10.3389/fpubh.2022.821150

**Published:** 2022-02-24

**Authors:** Jason N. Chen, Jeff A. Dennis, Julie A. St. John, Chwan-Li Shen

**Affiliations:** ^1^School of Medicine, Texas Tech University Health Sciences Center, Lubbock, TX, United States; ^2^Julia Jones Matthews Department of Public Health, Graduate School of Biomedical Sciences, Texas Tech University Health Sciences Center, Lubbock, TX, United States; ^3^Center of Excellence for Integrative Health, Lubbock, TX, United States; ^4^Julia Jones Matthews Department of Public Health, Graduate School of Biomedical Sciences, Texas Tech University Health Sciences Center, Abilene, TX, United States; ^5^Center of Excellence for Translational Neuroscience and Therapeutics, Lubbock, TX, United States; ^6^Department of Pathology, School of Medicine, Texas Tech University Health Sciences Center, Lubbock, TX, United States

**Keywords:** lifestyle medicine, education, compliance, behavior change, musculoskeletal disorder (MSD)

## Abstract

**Introduction:**

Approximately half of adult Americans suffer from musculoskeletal disorders (MSD). Significant risk factors for musculoskeletal disorders include poor diet, obesity, and insufficient physical activity. Studies show that lifestyle change education and interventions reduce MSD risk factors. However, little is known about the relationship between physician advice for behavior change and reported behavior change by MSD patients. This study explored the association between physician advice for lifestyle change and reported change in MSD patients, as well as the effects that patient education levels have on compliance.

**Methods:**

This study used data from the 2017 National Health Interview Survey, a nationally representative cross-sectional survey of non-institutionalized US adults. The research team limited analysis to adults who reported a limitation due to musculoskeletal problems (*n* = 2,672). Outcomes included physician recommendations to increase physical activity, reduce fat/calories, or lose weight, and whether they enacted these behavioral changes. Adjusted logistic regression models examined whether compliance with doctor's instructions differed by education level.

**Results:**

Adjusted models show patients advised to change physical activity, diet, and weight were more likely to report attempted behavior change. Education was positively associated with likelihood of complying with physician advice to increase physical activity. Among patients not advised to change behaviors by a physician, education was positively associated with current behavior change attempts.

**Conclusion:**

This study suggests that physician recommendations are relevant predictors of reported behavior change in individuals with MSD. Although education plays an important role in this association, the relationship is complex and multifaceted. Future studies should explore how compliance may be impacted by other factors, such as physician message type.

## Introduction

Musculoskeletal disorders (MSDs) affect ~124 million adult Americans, which accounts for one in every two adults, greater than the prevalence of cardiovascular and respiratory diseases combined (1). MSDs also have large economic implications, costing the US $213 billion in 2011 through lost productivity and more than $300 billion in 2009 through lost work earnings and health care costs (2). Pain and disability from MSDs are significant barriers to independent living in the community. Interventions need to include both clinical and population-based approaches to improve the prevention and treatment of MSDs for a better quality of life.

One of the most important areas for prevention and treatment is modifying risk factors (3). Numerous risk factors are associated with musculoskeletal conditions, including MSDs. Smoking, unhealthy diet, obesity, sleep deprivation, stress, and inactivity all contribute to a higher risk of developing MSDs (4). Minimizing the occurrence of these factors is well-accepted in reducing the risk and severity of MSDs as well as decreasing pain (5–7). However, long-term behavioral changes are essential for risk reduction and management of MSDs (8). Therefore, individuals need to focus on developing healthy choices with their physicians and maintaining them in their absence. Physician advice that is complemented with resources, such as community health programs, may improve prevention and risk factor management strategies (9).

Studies have shown that physicians play a significant role in educating and promoting healthier lifestyles in their patients (10, 11). Educational interventions in individuals with MSDs demonstrated effectiveness in reducing risk factors, improving productivity, and reducing pain and anxiety (12–14). However, non-compliance thwarts many effective treatment plans regardless of disease (15). Poor compliance is widespread, exhibited by ~30–50% of all patients and accounting for between $100 and $300 billion of avoidable health care costs (16). The relationship between physician advice for lifestyle change and MSD patient compliance has not been well-established. Appreciation of the factors that affect patients' decisions to perform and comply with advice is useful for healthcare providers and public health messaging to develop more efficient methods to maximize compliance and decrease risk factors in their patients. Further, an extension of compliance in the clinical setting can be applied to how people understand and respond to health initiatives in the community setting (17). In other words, the ability to understand the causes that help patients make better decisions with their physicians may also be a target for prevention strategies in the community.

One such factor is patient education level, which is a major social determinant of health essential for understanding and following a healthcare professional's advice (18–21). There is limited knowledge on how patient education levels are associated with compliance to healthcare providers' advice for lifestyle change to treat MSDs. Understanding this relationship may serve as a platform for further discoveries, such as determining how educational interventions affect lower educated patients (22). This may also aid the design of community outreach programs that help bring more attention to lifestyle choices and preventative measures. This study aimed to explore the association between patient behaviors for increasing physical activity, eating healthier, and enrolling in a weight loss program and physician advice for these lifestyle changes in MSD patients. Secondly, we examined the effects that background education level has on this compliance. We hypothesized a positive association between physician advice for behavior change and self-reported engagement in those behaviors and a positive association between education levels and compliance with physician's advice.

## Methods

This study analyzed data from the 2017 National Health Interview Survey (NHIS). The NHIS is a nationally representative cross-sectional survey of non-institutionalized US residents conducted yearly (23). Respondents aged 18 and up were used for this study, with data merged from the Person and Sample Adult files of the 2017 NHIS. The Sample Adult survey was administered to a subsample of the full NHIS sample, resulting in a sample size of 26,742. These respondents were asked, “What condition or health problem causes you to have difficulty with activities?” Those who answered that musculoskeletal or connective tissue problems caused such limitations represent the subpopulation of interest for the study (*n* = 2,672).

Outcomes of interest included respondent self-reports that a physician has instructed the person to participate in a weight loss program, increase physical activity, or reduce fat or calories. Additionally, the respondent self-reported if they are currently engaging in those three activities. Education was coded into four categories based on reported years of education completed: less than high school, high school diploma, some college, and college or more. Control variables included age group, race/ethnicity, gender, body mass index, marital status, and employment status.

The research team conducted the analysis using Stata 16.1. Chi square tests compared categorical variable distributions between education level and physician recommendations or health behavior efforts. The six binary outcomes in this study, including three self-reports of physician recommendation and three self-reports of current health behavior activity, had prevalence well above 10% in the population of interest. As such, these outcomes were not appropriate for logistic regression, which may overestimate effect size when the outcome occurs more than 10% of the time (24). Therefore, this study applied Poisson regression with robust error variance (calculated using population weights in Stata) to estimate the relative risk of each outcome (25, 26). Given that language of “risk” may be less palatable for discussing an outcome of favorable health behaviors, we report these as prevalence ratios (27). Although the prevalence of individuals saying they are currently engaged in a weight loss program is slightly <10% in the subpopulation (9.2%), prevalence ratios were reported for all outcomes for consistency. Population weights, using the “svy” command in Stata, were used to account for NHIS complex survey design. Statistical significance was set at alpha <0.05.

## Results

This study focuses on the 8.9% (*n* = 2,672) of 2017 adult NHIS respondents reporting that musculoskeletal or connective tissue problems cause limitations in activities. The average age for this subpopulation was older than the rest of the NHIS sample (56.4 years compared to 46.5 years, respectively). The MSD subsample was also more likely to be female than the rest of the NHIS sample (61.1 vs. 50.8%, respectively). The sample was also disproportionately non-Hispanic white relative to the rest of the NHIS sample (72.1 vs. 63.4%, respectively).

The proportion of adult respondents with MSD who reported being told by a physician to reduce fat and calories in their diet significantly differed by education level, where 48.73% with less than high school education reported receiving this advice, compared to 33.07% with a college degree or higher (Table 1). Education level was not associated with physician advice to increase physical activity or participate in a weight loss program.

**Table 1:** Education effects on the proportions of adults with musculoskeletal disorders receiving physician advice for behavior change and reporting performing those changes.

	** < HS**	**HS diploma**	**Some college**	**College or more**		
	***N* (%)**	***N* (%)**	***N* (%)**	***N* (%)**	Chi-square^*^	***p*-value**
Told to increase physical activity, past 12 m	175 (51.35)	360 (50.06)	416 (50.27)	329 (47.58)	18.140	0.767
Told to reduce fat/calories in diet, past 12 m	164 (48.73)	293 (40.81)	329 (40.71)	231 (33.07)	261.883	0.001
Told to participate in weight loss program, past 12 m	49 (15.14)	86 (13.14)	117 (15.02)	86 (12.07)	36.416	0.533
Currently increasing physical activity	181 (49.78)	408 (55.36)	502 (61.14)	463 (65.83)	322.767	0.001
Currently reducing fat/calories in diet	179 (54.87)	399 (53.90)	516 (60.92)	406 (58.67)	94.078	0.114
Currently participating in weight loss program	21 (7.71)	46 (6.14)	87 (10.74)	81 (11.67)	170.282	0.024

* *Chi-square tests difference in educational attainment and Yes/No answer to engaging in each of the above categories*.

Education and self-reports of current efforts to increase physical activity were positively associated, with 49.78% of adults with less than a high school degree reporting increased physical activity attempt, compared to 65.83% of adults with college or greater degrees (Table 1). A similar trend was true for respondents participating in a weight loss program, where 7.71% with less than a high school degree participated compared to 11.67% of college graduates. Education was not associated with current efforts to reduce fat or calories.

Adjusted prevalence ratios predicting physician recommendations for behavior change show few significant associations (Table 2). Education was not associated with physician recommendations except in the case of calorie reduction, where those with a college degree exhibited a lower prevalence of being told to reduce calories compared to those with less than a high school degree (OR = 0.78, 95% CI−0.64–0.95).

**Table 2:** Adjusted prevalence ratios predicting physician recommendations for behavior change in individuals reporting musculoskeletal disorders.

	**Told to increase physical activity**		**Told to reduce calories**		**Told to participate in weight loss program**	
	**PR (se)**	**sig**	**PR (se)**	**sig**	**PR (se)**	**sig**
Female	1.19 (0.06)	^***^	1.05 (0.06)		0.83 (0.1)	
Age (ref = 18–29)						
30–39	0.77 (0.1)		0.88 (0.16)		1.01 (0.42)	
40–49	0.9 (0.11)		0.98 (0.15)		1.13 (0.43)	
50–64	1.07 (0.12)		1.21 (0.17)		1.34 (0.45)	
65+	0.99 (0.11)		1.17 (0.17)		1.02 (0.35)	
Race/ethnicity (ref = NH white)						
Hispanic	1.17 (0.1)		1.25 (0.11)	^*^	1.53 (0.34)	
NH black	1.03 (0.08)		1.07 (0.09)		1.27 (0.23)	
Other	1.32 (0.16)	^*^	1.38 (0.19)	^*^	1.22 (0.42)	
Education (ref = Less than HS)						
HS diploma	1.02 (0.08)		0.89 (0.07)		0.93 (0.19)	
Some college	1.00 (0.08)		0.85 (0.07)		1.01 (0.2)	
College or more	1.03 (0.09)		0.78 (0.08)	^*^	1.01 (0.21)	
Married	1.14 (0.05)	^**^	1.02 (0.06)		1.05 (0.13)	
Employed	0.93 (0.06)		0.99 (0.06)		0.93 (0.13)	
BMI (ref = 18.5 to <25)						
Overweight	1.68 (0.16)	^***^	2.08 (0.3)	^***^	2.13 (0.82)	
Obese	2.29 (0.2)	^***^	3.68 (0.48)	^***^	7.09 (2.5)	^***^
*N*	2,541		2,542		2,543	

* *p < 0.05;*

** *p < 0.01;*

*** *p < 0.001*. *PR, prevalence ratio; se, standard error; NH, non-Hispanic; ref, reference group; BMI, body mass index; HS, high school; sig, significance*.

**Figure 1:**
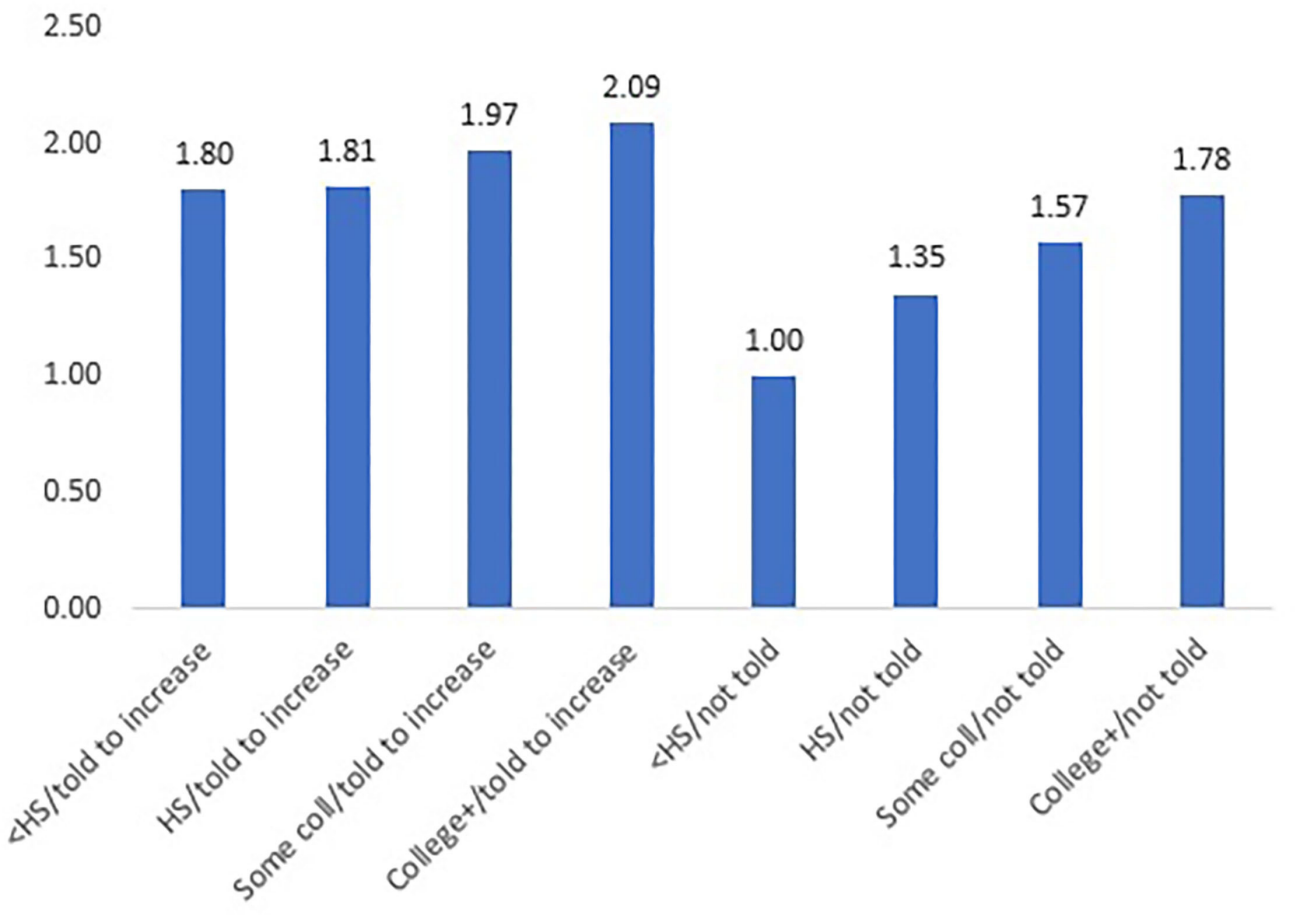
Prevalence ratios of attempting to increase physical activity, by education and whether doctor advised to increase physical activity. < HS, less than high school diploma; HS, high school diploma; Some coll, some college; College+, college degree or higher; Prevalence ratio for each bar derived from interaction of poisson regression coefficients between doctor's recommendation to increase physical activity and education level; Reference group, less than a HS diploma who were not told to increase physical activity.

Education was positively associated with alignment of behavior change efforts and self-reported physician recommendations to increase physical activity (Table 3). Significant interaction effects demonstrate that efforts to increase physical activity vary by education level and self-reported physician recommendations (Figure 1). There was also a positive trend between education level and the likelihood of attempting physical activity among those who were not advised to do so by a physician. The physician recommendation group exhibited overall higher efforts to increase physical activity and narrower disparities between high and low education groups.

**Table 3:** Adjusted prevalence ratios predicting current behavior change efforts in individuals reporting musculoskeletal disorders.

	**Currently increasing physical activity**		**Currently reducing calories**		**Currently in weight loss program**	
	**PR (se)**	**sig**	**PR (se)**	**sig**	**PR (se)**	**sig**
Female (ref = male)	0.09 (0.05)		0.12 (0.05)	^**^	0.31 (0.20)	^*^
Age (ref = 18–29)						
30–39	−0.08 (0.08)		0.16 (0.14)		−0.01 (0.48)	
40–49	−0.18 (0.08)	^*^	0.26 (0.15)	^*^	0.23 (0.52)	
50–64	−0.08 (0.07)		0.23 (0.13)	^*^	0.84 (0.88)	^*^
65+	−0.23 (0.06)	^**^	0.18 (0.13)		0.3 (0.53)	
Race/ethnicity (ref = NH white)						
Hispanic	0.05 (0.07)		0.12 (0.07)		−0.54 (0.17)	
NH black	0.08 (0.06)		0 (0.06)		−0.42 (0.19)	
Other	0.09 (0.11)		0.23 (0.12)	^*^	0.0 (0.30)	
Education (ref = Less than HS)						
HS diploma	0.30 (0.22)		0.24 (0.19)		−0.30 (0.20)	
Some college	0.45 (0.23)	^**^	0.50 (0.25)	^***^	0.19 (0.30)	
College or more	0.57 (0.27)	^***^	0.51 (0.24)	^***^	0.26 (0.32)	
Married	−0.01 (0.04)		0.06 (0.04)		−0.09 (0.12)	
Employed	0.02 (0.04)		0.09 (0.05)		0.33 (0.22)	^*^
BMI (ref = 18.5 to <25)						
Overweight	0.17 (0.08)	^**^	0.25 (0.09)	^***^	0.58 (0.47)	^*^
Obese	0.12 (0.07)		0.28 (0.08)	^***^	0.66 (0.49)	^**^
Told to increase physical activity	0.59 (0.29)	^***^				
Told to reduce fat/calories			0.90 (0.35)	^***^		
Told to participate in weight loss					1.74 (0.91)	^***^
Interaction terms						
HS diploma X told to increase physical activity	−0.29 (0.14)					
Some college X told to increase physical activity	−0.36 (0.12)	^*^				
College degree X told to increase physical activity	−0.43 (0.11)	^*^				
HS diploma X told to reduce fat/calories			−0.26 (0.13)			
Some college X told to reduce fat/calories			−0.47 (0.10)	^**^		
College degree X told to reduce fat/calories			−0.53 (0.09)	^***^		
N	2,541		2,540		2,543	

* *p < 0.05;*

** *p < 0.01;*

*** *p < 0.001*. *PR, prevalence ratio; se, standard error; NH, non-Hispanic; ref, reference group; BMI, body mass index; HS, high school; sig, significance*.

**Figure 2:**
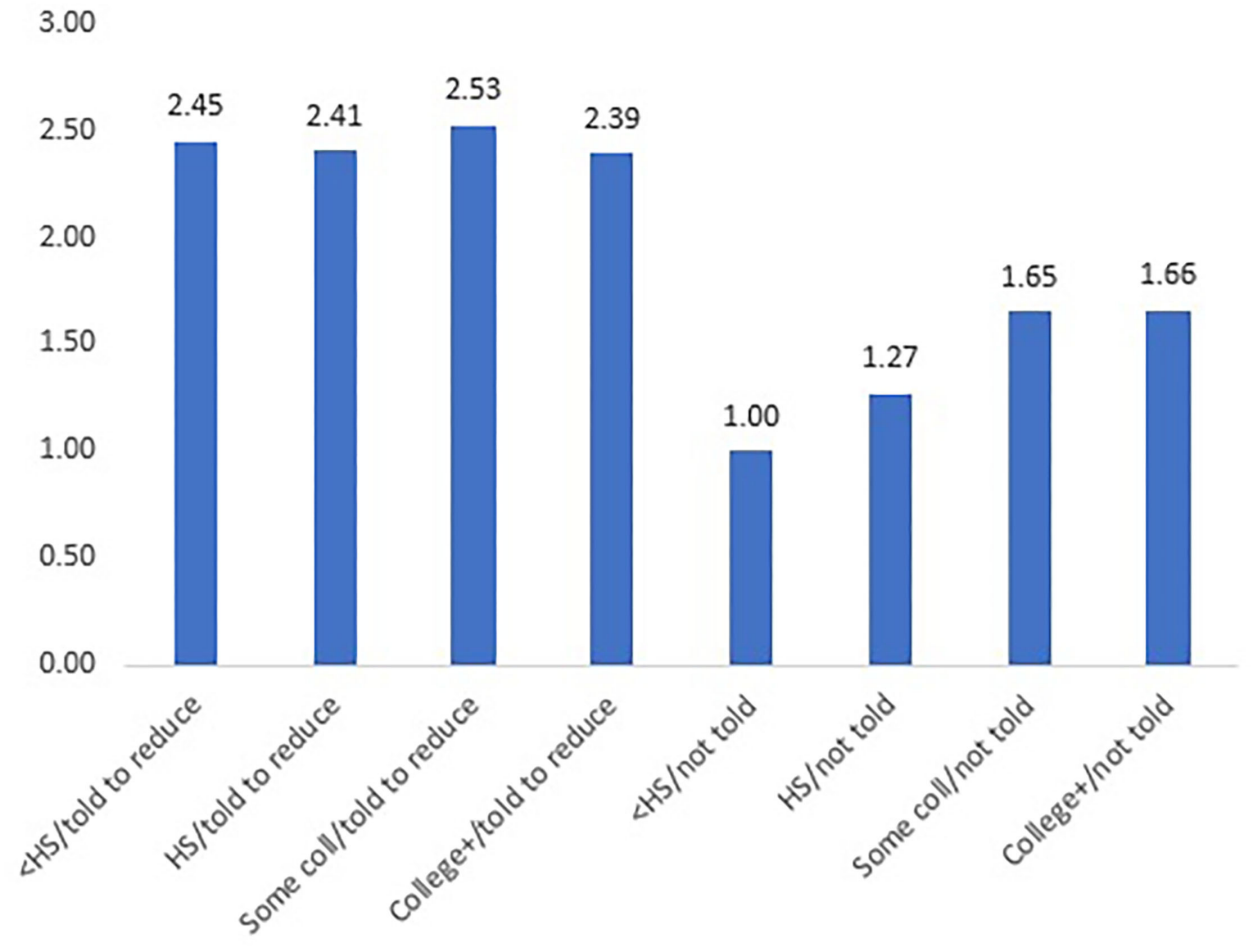
Prevalence ratios of attempting to reduce calories, by education and whether doctor advised to decrease calories. < HS, less than high school diploma; HS, high school diploma; Some coll, some college; College+, college degree or higher; Prevalence ratio for each bar derived from interaction of poisson regression coefficients between doctor's recommendation to reduce calories and education level; Reference group, less than a HS diploma who were not told to reduce calories.

Education was also positively associated with self-reported attempts to reduce calories, although the interaction effects demonstrate that this relationship was primarily rooted in the group not told to reduce calories (Table 3). The MSD group told to reduce calories by a physician exhibited no meaningful differences by education level (Figure 2). Interactions between education and physician recommendations for participation in a weight loss program were not statistically significant and were dropped from the final model. Individuals who self-reported that their physician recommended a weight loss program were 1.74 times more likely to report participating in one.

## Discussion

This cross-sectional study highlighted the modest, yet meaningful, role of physician advice for behavior changes among MSD patients. Those who were advised to exercise more, participate in weight loss programs, and reduce calories and fat were more likely to do so compared to patients who were not. These findings are consistent with previous research addressing non-musculoskeletal patient compliance to similar health promoting behavior changes (10, 11, 28). For increasing physical activity and reducing calories, physician recommendation for behavior change was an important factor that attenuated educational disparities in these behaviors.

Physicians have a vital role in counseling patients and have the opportunity at each encounter to educate their patients. In a study using National Health and Nutrition Examination Survey, Pool et al. reported that patients who had their obesity addressed by their physicians were two times more likely to report a 5% weight loss compared to patients whose physicians did not (29). The current study shows that about half of respondents report that their physician has told them to increase physical activity, whereas about 12–15% report their physician has recommended a weight loss program. This suggests that some physicians may be missing opportunities to advise MSD patients on the benefits of lifestyle changes or patients are not receiving messages that are conveyed to them. More comprehensive dissemination of these recommendations can produce significant risk factor reductions and thus serves as an important method for helping prevent and treat MSDs (5–7, 30).

Several studies have supported that educational interventions mitigating risk factors help prevent workers from developing MSDs (12–14). The current study supports the idea that the educational baseline of patients has an impact on compliance with physician advice. However, this association is complex and further investigation is needed to understand the factors that may cause the positive trend between educational background and compliance. For example, if misunderstanding of advice or patient distrust with their doctors are different among the education groups, which may impact compliance (31, 32). More targeted approaches based on education level may increase the efficacy of physician recommendations for behavioral change.

Patient perception also complicates compliance relationships. Whereas, healthcare providers may prioritize compliance, patients may have higher priorities such as controlling symptoms, preventing medical emergencies, maintaining financial stability, or enjoying a quality lifestyle (33). Lifestyle behavior change recommendations for patients suffering especially from chronic diseases may not all align with their best interests (33). Variations in patient perception for what is best for them may help explain why there was a significant impact of education on increasing physical activity and decreasing fat/calories, but not enrolling in a weight loss program. Further investigation is needed to understand the patient perspective on following advice and how educational attainment may impact how patients perceive what is best for them. As our models indicate, understanding reported patient compliance with physician recommendation may also be impacted by gender, race/ethnicity, and age. Previous investigations have indicated that men exhibit a lower degree of medical compliance compared to women (34). Additionally, race/ethnicity analyses showed that European Americans reported significantly better compliance than Asian Americans and Hispanics (34). Such factors add to the complexity of understanding patient perception and thus compliance.

This study is limited by its survey-based self-report nature, and therefore, recall bias may be possible in relation to patient recall of doctor's recommendations and patient reports of health behaviors. The cross-sectional nature of this study does not allow us to establish causation among reported advice, reported behavior change, or education level, as we do not know the timing of the recommendation relative to the report of attempted behavior change. Additionally, other factors such as age, race/ethnicity, and gender may impact the efficacy of behavior change recommendations and deserve exploration in future studies. Lastly, patients may have received more than one behavior change advice. Separating the three advice categories such that there was no overlap in advice given to subjects was not possible due to issues with sample size. The strength of this study is that the subpopulation of individuals whose musculoskeletal symptoms cause difficulty with daily activities is derived from a large, nationally representative sample of non-institutionalized US adults. As such, the sample may be more representative of US adults broadly experiencing such symptoms, including those actively being treated and those who have not or will not seek treatment for their symptoms.

The findings suggest that physician advice for risk factor management to prevent MSDs and improve patient outcomes should not be discounted. Doctors should strive to provide their patients with a sufficient understanding of the benefits of a regimen and how to follow the proposed regimen. This compliance may also apply to beyond the clinic. Spreading awareness of modifying behaviors to reduce risk factors in the community may greatly reduce the prevalence of musculoskeletal conditions. The educational background of a patient should also be considered when treating for MSD, and different approaches may be needed when addressing differently educated patients to maximize their compliance and understanding of advice.

## Conclusion

Physician advice for lifestyle behavior change increased the likelihood for MSD patients to increase physical activity, decrease fat and calories, and participate in a weight loss program. Additionally, education differences were attenuated when patients were told by their physician to increase physical activity or decrease fat and calories. This demonstrates the importance of physician communication with their patients for advocating healthy behaviors, regardless of education level. Compliance with advice was affected by education for increasing physical activity. This relationship is complex and warrants future investigation for possible explanations.

## Data Availability Statement

Publicly available datasets were analyzed in this study. This data can be found at: https://www.cdc.gov/nchs/nhis/nhis_2017_data_release.htm.

## Ethics Statement

Ethical review and approval was not required for the study on human participants in accordance with the local legislation and institutional requirements. Written informed consent for participation was not required for this study in accordance with the national legislation and the institutional requirements.

## Author Contributions

JC: conceptualization, methodology, validation, investigation, writing—original draft, and writing—review and editing. JD: conceptualization, methodology, formal analysis, investigation, writing—original draft, and writing—review and editing. JS: conceptualization and writing—review and editing. C-LS: conceptualization, writing—review and editing, and supervision. All authors contributed to the article and approved the submitted version.

## Conflict of Interest

The authors declare that the research was conducted in the absence of any commercial or financial relationships that could be construed as a potential conflict of interest.

## Publisher's Note

All claims expressed in this article are solely those of the authors and do not necessarily represent those of their affiliated organizations, or those of the publisher, the editors and the reviewers. Any product that may be evaluated in this article, or claim that may be made by its manufacturer, is not guaranteed or endorsed by the publisher.
